# Real-life effects of nudging and pricing strategies in the supermarket to promote healthy diets

**DOI:** 10.1093/eurpub/ckac129.550

**Published:** 2022-10-25

**Authors:** JM Stuber, JD Mackenbach, J Lakerveld, JWJ Beulens

**Affiliations:** Epidemiology and Data Science, Amsterdam UMC, Amsterdam, Netherlands

## Abstract

**Background:**

Unhealthy dietary patterns pose a major public health challenge. Individual-level efforts to promote heathy diets (e.g. nutrition education) have limited effect on the long term. Context-specific interventions focussing on point-of-purchase may create opportunities for sustainable dietary changes. We evaluated real-life effects of nudging and pricing strategies in supermarkets on dietary intake.

**Methods:**

In this parallel cluster-randomised controlled trial, we randomized 12 Dutch supermarkets in socially deprived neighbourhoods to a control group (n = 6), or intervention group (n = 6). Healthy food nudges were implemented in intervention stores across 13 food groups, combined with healthy product price decreases and unhealthy product price increases. Eligible participants were aged 30-80 years and regular shoppers at participating stores. The primary outcome was dietary guideline adherence measured via an index score (0-150), at baseline and after 3, 6 and 12 months. Secondary outcomes included parameters of cardiometabolic health (HbA1c, lipid profile, and waist circumference), the percentage of healthy food purchases in the supermarket, socio-cognitive factors, and supermarket customer satisfaction. Effects were analysed with linear mixed models.

**Results:**

This study included 173 participants from intervention clusters and 220 from control clusters. Preliminary evaluation of the 3-month follow-up data revealed no effectiveness of the nudging and pricing strategies compared to the control supermarkets in terms of dietary guideline adherence (β -0.8, 95%CI -4.2; 2.7). Results on all outcomes will be available at time of the conference.

**Conclusions:**

This novel supermarket trial is the first to evaluate real-life long-term effects of nudging and pricing strategies based on a comprehensive set of study outcomes and using a strong methodological design. Findings can direct future design of context-specific interventions focussing on the promotion of healthy diets.

**Key messages:**

• Context-specific interventions focussing on point-of-purchase may create opportunities for sustainable dietary changes.

• Findings from this supermarket trial testing real-life and long-term effects of nudging and pricing strategies can direct future design of context-specific interventions focussing on healthy diets.

